# Supplementary material for: Hippocampal and Reticulo-Thalamic Parvalbumin Interneurons and Synaptic Re-Organization during Sleep Disorders in the Rat Models of Parkinson’s Disease Neuropathology
Source: Int J Mol Sci. 2021 Aug 19;22(16):8922. doi: 10.3390/ijms22168922 (PMC8396216; doi:10.3390/ijms22168922)
Supplement: Supplementary file 1 [file ijms-22-08922-s001.zip › ijms-1328888-supplementary.pdf]

Hippocampal and Reticulo-Thalamic Parvalbumin Interneurons and Synaptic Re-Organization during Sleep Disorders in the Rat Models of Parkinson's Disease Neuropathology

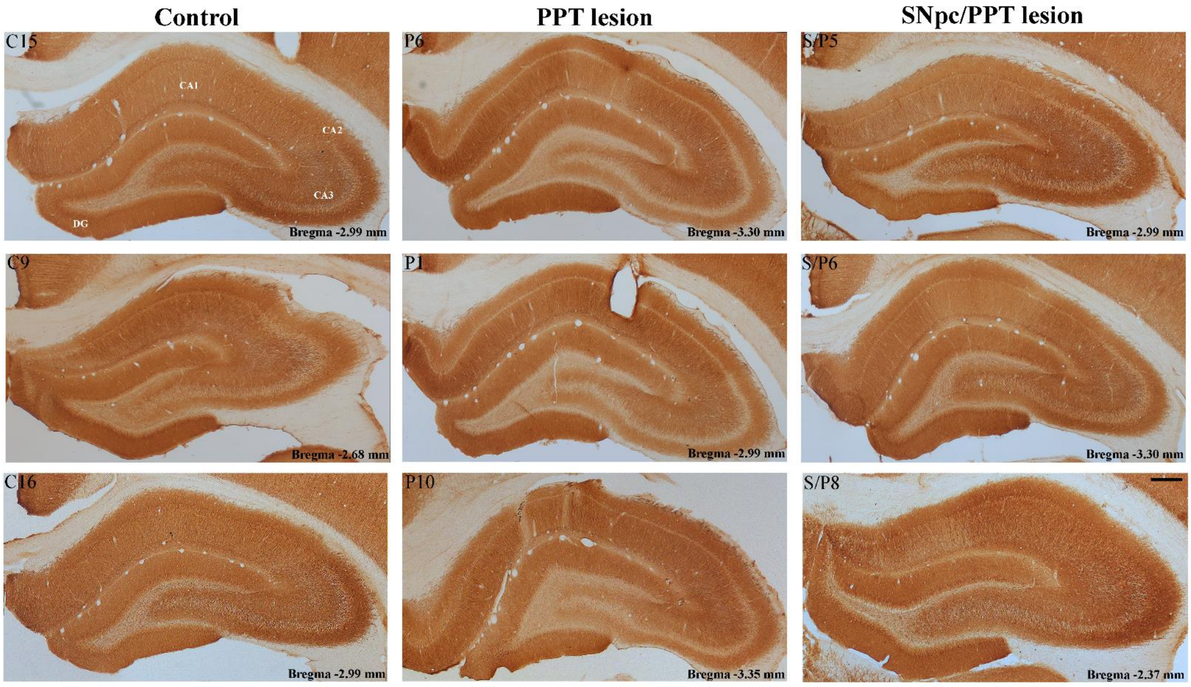

Figure S1. Alteration of the hippocampal MAP2 immunostaining in the distinct rat models of PD neuropathology. The MAP2 immunoreactivity within the hippocampus of the control rats (Control, C15, C9, C16), the rats with PD cholinopathy (PPT lesion, P6, P1, P10) and the hemiparkinsonian rats with PD cholinopathy (SNpc/PPT lesion, S/P5, S/P6, S/P8). DG – dentate gyrus. CA1 – CA1 field of the hippocampus; CA2 – CA2 field of the hippocampus; CA3 – CA3 field of the hippocampus. Scale bar is 400 µm.

MAP2 immunoreactivity was suppressed in the rats with PD cholinopathy, but it was enhanced in the hemiparkinsonian rats with PD cholinopathy within the DG granular and

polymorphic cell layers, and in the pyramidal cell layer and stratum radiatum of the hippocampal CA3 region vs. the control.

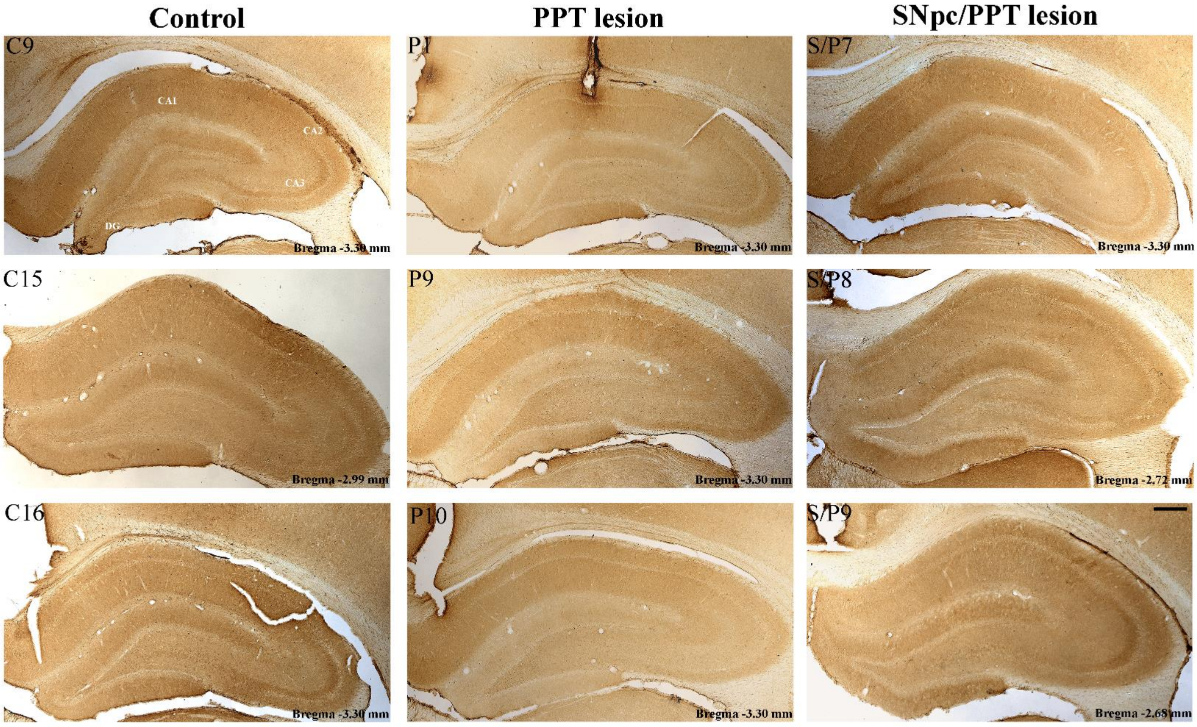

Figure S2. Alteration of the hippocampal PSD-95 immunostaining in the distinct rat models of PD neuropathology. The PSD-95 immunoreactivity within the hippocampus of the control rats (Control, C9, C15, C16), the rats with PD cholinopathy (PPT lesion, P1, P9, P10) and the hemiparkinsonian rats with PD cholinopathy (SNpc/PPT lesion, S/P7, S/P8, S/P9). DG – dentate gyrus. CA1 – CA1 field of the hippocampus; CA2 – CA2 field of the hippocampus; CA3 – CA3 field of the hippocampus. Scale bar is 200 μm.

Whereas the PSD-95 immunoreactivity was suppressed in the rats with PD cholinopathy within the granular and molecular cell layers of the DG and within the pyramidal cell layer and stratum radiatum of the hippocampal CA3 region vs. the control rats, the PSD-95 immunoreactivity was enhanced in the hemiparkinsonian rats with PD cholinopathy within the granular and molecular cell layers of the DG vs. the control rats.

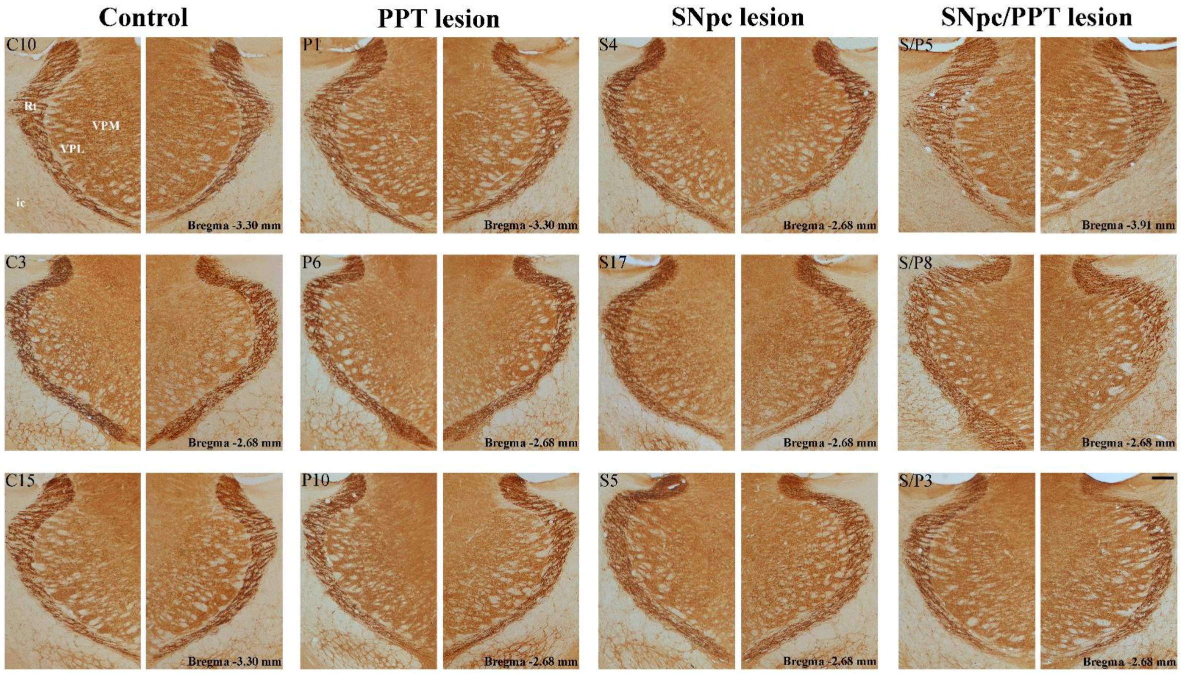

Figure S3. Alteration of PV immunostaining within the RT in the distinct rat models of PD neuropathology. The PV immunoreactivity within the RT of the control rats (Control, C10, C3, C15), the rats with PD cholinopathy (PPT lesion, P1, P6, P10), the hemiparkinsonian rats (SNpc lesion, S4, S17, S5) and the hemiparkinsonian rats with PD cholinopathy (SNpc/PPT lesion, S/P5, S/P8, S/P3). Rt – reticular nucleus; VPM – ventral posteromedial thalamic nucleus; VPL – ventral posterolateral thalamic nucleus; ic – internal capsule. Scale bar is 200 μm.

PV immunoreactivity was reduced within the RT of the hemiparkinsonian rats with PD cholinopathy (SNpc/PPT lesion) ipsilaterally to the combined SNpc/PPT lesion (the right RT) vs. the contralateral RT (the left RT), and vs. the control rats, the rats with PD cholinopathy and the hemiparkinsonian rats. The visible defects of the PV immunoreactivity within the RT of the hemiparkinsonian rats with PD cholinopathy spread from 0.64 to 1.84 mm.

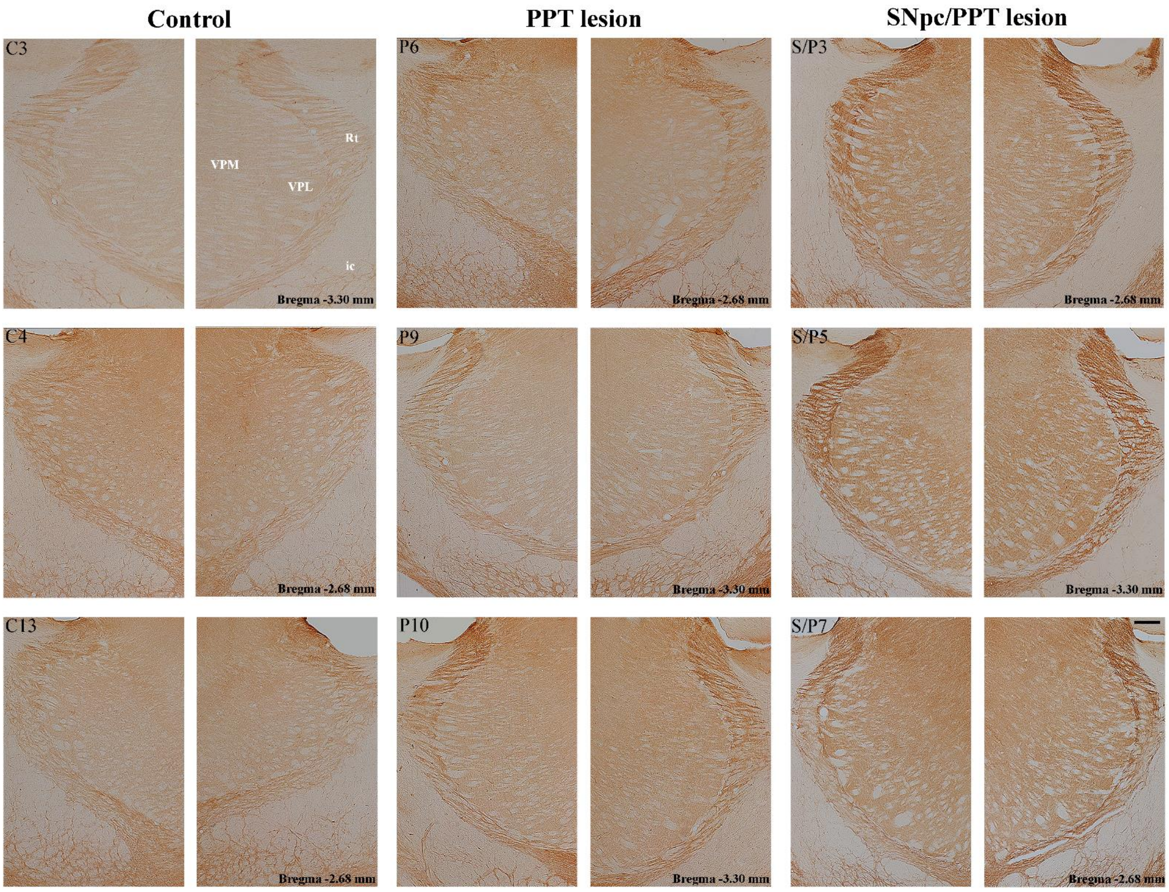

Figure S4. Alteration of the MAP2 immunostaining within the RT in the distinct rat models of PD neuropathology. The MAP2 immunoreactivity within the RT of the control rats (Control, C3, C4, C13), the rats with PD cholinopathy (PPT lesion, P6, P9, P10) and the hemiparkinsonian rats with PD cholinopathy (SNpc/PPT lesion, S/P3, S/P5, S/P7). Rt – reticular nucleus; VPM – ventral posteromedial thalamic nucleus; VPL – ventral posterolateral thalamic nucleus; ic – internal capsule. Scale bar is 200 µm.

MAP2 immunoreactivity was enhanced within the RT of the hemiparkinsonian rats with PD cholinopathy (SNpc/PPT lesion) ipsilaterally to the combined SNpc/PPT lesion (the right RT) vs. the contralateral RT (the left RT), vs. the control rats and the rats with PD cholinopathy.

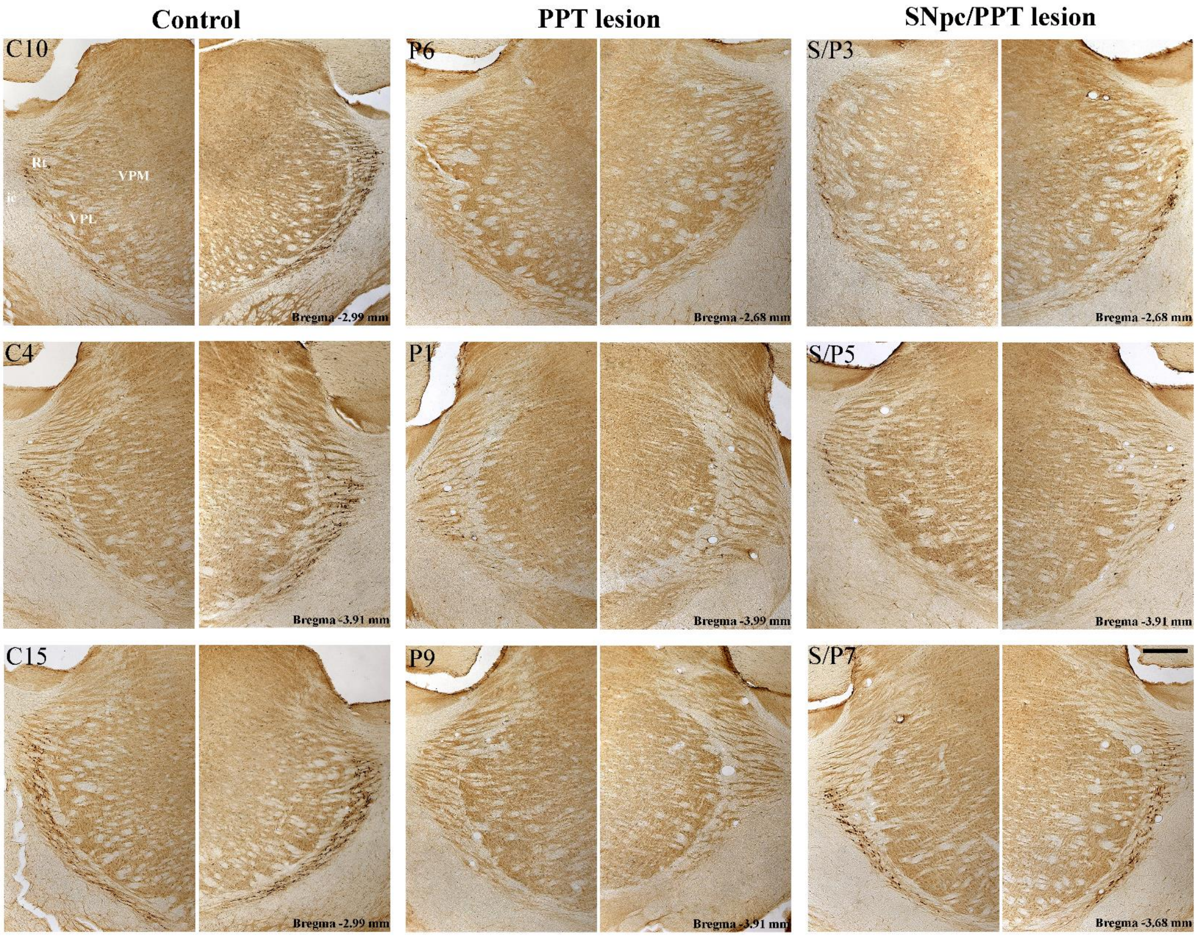

Figure S5. Alteration of the PSD-95 immunostaining within the RT in the distinct rat models of PD neuropathology. The PSD-95 immunoreactivity within the RT of the control rats (Control, C10, C4, C15), the rats with PD cholinopathy (PPT lesion, P6, P1, P9) and the hemiparkinsonian rats with PD cholinopathy (SNpc/PPT lesion, S/P3, S/P5, S/P7). Rt – reticular nucleus; VPM – ventral posteromedial thalamic nucleus; VPL – ventral posterolateral thalamic nucleus; ic – internal capsule. Scale bar is 200 µm.

The PSD-95 immunoreactivity was suppressed within the RT of the rats with PD cholinopathy, but it was similar to the control level in the hemiparkinsonian rats with PD cholinopathy.
